# Regulation of ecdysone production in *Drosophila* by neuropeptides and peptide hormones

**DOI:** 10.1098/rsob.200373

**Published:** 2021-02-17

**Authors:** Jade R. Kannangara, Christen K. Mirth, Coral G. Warr

**Affiliations:** ^1^ School of Biological Sciences, Monash University, Clayton, Victoria 3800, Australia; ^2^ Tasmanian School of Medicine, University of Tasmania, Hobart, Tasmania 7000, Australia

**Keywords:** neuropeptides, ecdysone, signalling, development, growth, *Drosophila melanogaster*

## Abstract

In both mammals and insects, steroid hormones play a major role in directing the animal's progression through developmental stages. To maximize fitness outcomes, steroid hormone production is regulated by the environmental conditions experienced by the animal. In insects, the steroid hormone ecdysone mediates transitions between developmental stages and is regulated in response to environmental factors such as nutrition. These environmental signals are communicated to the ecdysone-producing gland via the action of neuropeptide and peptide hormone signalling pathways. While some of these pathways have been well characterized, there is evidence to suggest more signalling pathways than has previously been thought function to control ecdysone production, potentially in response to a greater range of environmental conditions. Here, we review the neuropeptide and peptide hormone signalling pathways known to regulate the production of ecdysone in the model genetic insect *Drosophila melanogaster*, as well as what is known regarding the environmental signals that trigger these pathways. Areas for future research are highlighted that can further contribute to our overall understanding of the complex orchestration of environmental, physiological and developmental cues that together produce a functioning adult organism.

## Graphical abstract

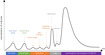

## 1. Introduction

Most animals go through several developmental stages during their life cycle. For example, after hatching most insects go through multiple nymphal or larval stages before initiating metamorphosis into adults. To maximize animal fitness and survival, the rate and duration of each of these developmental stages must be precisely regulated in response to environmental conditions.

Across a broad range of animals, steroid hormones are important regulators of developmental time. In insects, the steroid hormone ecdysone regulates when larvae moult, initiate metamorphosis and differentiate adult structures. As final body size is a product of both the rate and duration of the growth phase [1], by regulating developmental time ecdysone also impacts final body size [2]. The growth phase occurs either between the hatching of a nymph and the adult moult in hemimetabolous insects, or between the hatching of a larva and the onset of metamorphosis in holometabolous insects. Once insects have reached the adult stage their rigid adult exoskeleton prevents further growth. For this reason, changing development time in the nymphal or larval stages can have profound effects on the final adult size.

Many of the developmental processes that ecdysone regulates are sensitive to a number of different environmental conditions, such as the photoperiod, nutrition, oxygen levels and temperature [3–9]. Studies in recent years have indicated that responses to these environmental conditions are mediated by neuropeptides and peptide hormones that regulate ecdysone synthesis. However, to date researchers are yet to match the full range of environmental conditions known to modify the timing and quantity of ecdysone synthesis with the peptide signals they induce.

**Figure 1.**
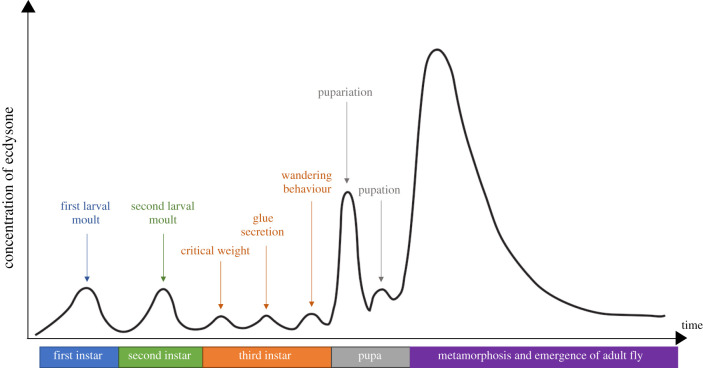
Several pulses of ecdysone are produced during *Drosophila* development, and each pulse correlates with a particular developmental transition. These pulses of ecdysone trigger larval moults, as well as pupariation and pupation [2]. In addition, ecdysone also activates other developmental checkpoints and behaviours such as critical weight, glue secretion and wandering behaviour [2,10].

The fruit fly, *Drosophila melanogaster,* is an excellent model insect in which to identify the full range of peptide signals that regulate ecdysone synthesis. This is because peptide signalling pathways can be easily manipulated in the fly, including in a tissue- and time-specific manner, which is extremely useful given many of these peptide hormones regulate multiple biological processes. *Drosophila* undergoes three larval moults, initiates metamorphosis in the pre-pupal stage, continues adult development in the pupal stage and finally emerges as an adult. The duration and rate of growth of the larval stages, as well as the initiation of pupariation, are under the control of carefully timed pulses of ecdysone (figure 1).

A pulse of ecdysone is produced just before each larval moult (for review, see [11]), and these pulses trigger the expression of the genes that are required for the moults to occur. Three additional pulses of ecdysone are also produced within the third larval instar [10]. The first of these pulses controls a developmental checkpoint known as critical weight [10,12]. Once the critical weight is reached, starvation can no longer delay the onset of metamorphosis [5–7,12,13]. A second small pulse of ecdysone initiates the onset of glue production [10]. This glue will later be used to stick the pupal case formed at pupariation to a given surface [14]. Later in the third larval instar, a small pulse of ecdysone is produced that induces wandering behaviour, where the animal stops feeding and begins searching for a suitable location to begin pupariation [10]. Finally, the last pulse of ecdysone produced controls the onset of pupariation, signalling the beginning of pre-pupal and then pupal development [10]. If a pulse of ecdysone is absent or incorrectly produced, the animal suffers developmental defects such as delays in development, which can be accompanied by changes in final body size, or even lethality [3,5–7,15]. This demonstrates the importance of this hormone in regulating these developmental transitions and for overall growth.

In addition to its role in regulating developmental timing and body size, ecdysone is also known to play other important roles throughout the life cycle. For example, ecdysone is synthesized during mid to late embryogenesis in *Drosophila* and other insects [16], correlating with the morphogenetic changes associated with the development of the first instar larva [17] and with cuticle formation [18]. Ecdysone also plays a role in the growth and patterning of developing adult tissues such as the wing imaginal discs and the neuroblasts of the central nervous system, in both *Drosophila* [19–22] and the tobacco hornworm, *Manduca sexta* [20,23–25]. During metamorphosis, ecdysone is required for the eversion and proliferation of cells of the imaginal discs, for apoptosis of larval cells, and for restructuring the nervous system (for review, see [2,26]). In the adult fly, ecdysone influences egg development in the female germline [27,28]. While all the roles ecdysone plays throughout the life cycle are important, this review focuses on the role ecdysone plays in regulating development during the larval stages and the environmental control of ecdysone in this context.

## 2. Ecdysone production and secretion

**Figure 2.**
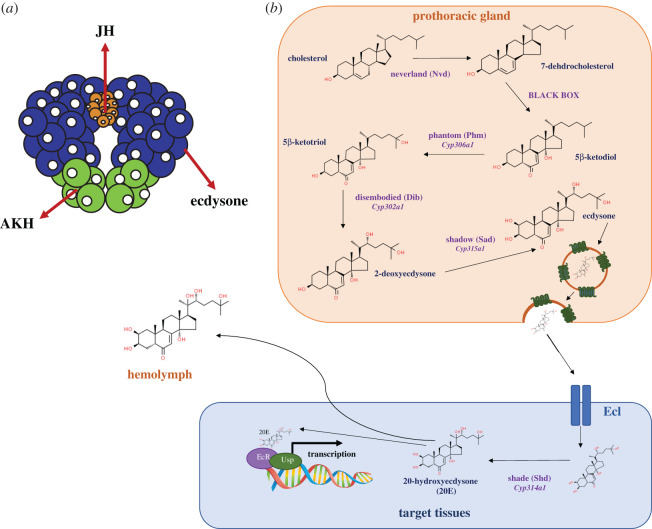
The prothoracic gland produces and secretes ecdysone. The ring gland comprises the corpora allata (CA) (orange), which produces and secretes Juvenile Hormone (JH), the corpora cardiaca (CC) (green), which produces and secretes Adipokinetic hormone (AKH), and the prothoracic gland (PG) (purple), which produces and secretes pulses of ecdysone throughout development. (*b*) Ecdysone is synthesized from dietary cholesterol by several ecdysone biosynthetic enzymes, including *phantom, shade*, *disembodied, shadow* and *neverland* (for review, see [30]). The ‘Black box' genes are a group of genes that have not been characterized, but are predicted to be responsible for converting 7-dehydrocholesterol to 5β-ketodiol [31,32]. Ecdysone is then secreted from the PG via vesicle exocytosis into the haemolymph [33], where it can enter target tissues via Ecdysone Importer (EcI) [34,35]. It is then converted to its active form, 20E, by Shade and can bind to the nuclear receptor complex, Ecdysone Receptor (EcR) and Ultraspiracle (Usp), to activate the downstream transcription factors that promote developmental transitions [36].

In the *Drosophila* larva, ecdysone is produced in and secreted from paired endocrine glands known as the prothoracic glands (PG). The PGs are part of a composite gland known as the ring gland, which also comprises the corpora allata and corpora cardiaca (for review, see [29]), which produce juvenile hormone and adipokinetic hormone (AKH), respectively (figure 2*a*). Ecdysone is synthesized in the PG from dietary cholesterol by a group of P450 enzymes collectively known as the ‘Halloween genes' (figure 2*b*; for review, see [30]). The Halloween genes act in a sequential manner to convert cholesterol and other sterols into ecdysone [37]. Ecdysone is then actively secreted from the PG into the haemolymph. Ecdysone has been shown to enter cells via facilitated diffusion through a membrane transporter, Ecdysone Importer (EcI) [34,35], although it remains possible that it also enters cells via free diffusion. Once in the cytoplasm, it is converted to its active form, 20-hydroxyecdysone (20E) by the enzyme Shade [38]. Throughout the rest of this review, the term ‘ecdysone' will be used to refer to both the inactive precursor and the active form 20E. Ecdysone activates the nuclear ecdysone receptor complex, which is a heterodimer of two proteins, Ecdysone Receptor and Ultraspiracle, in target tissues. The ecdysone receptor complex binds to ecdysone-response elements and triggers the expression of ecdysone target genes that promote developmental transitions [36]. It is also likely that after being converted to its active form in target tissues ecdysone is released back into the haemolymph. This is because the active form of ecdysone has been detected in the haemolymph, with peaks identified just before moulting and metamorphosis [37,39].

**Figure 3.**
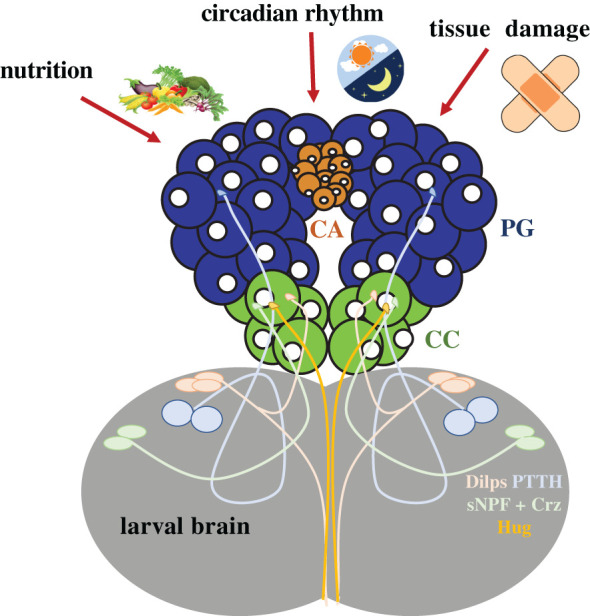
Examples of known neuropeptide-secreting neurons that innervate the *Drosophila* ring gland. The PG produces and secretes ecdysone in response to environmental and physiological stimuli. These environmental signals also trigger the secretion of peptides onto the PG. Sources of such peptides include the PTTH-producing neurons, which innervate the PG. Additionally, the insulin-producing cells secrete the Dilps, and Dilp-producing neurons innervate the CC [41]. Furthermore, *short neuropeptide f* (*sNPF*)-, *corazonin* (*Crz)-* and *hugin* (*Hug*)-expressing neurons also innervate the CC [42–44], although whether these function in the PG is unknown.

The PG can be thought of as a sensor, responding to signals from the external environment to ensure that ecdysone is produced at the right time, and in the right amount, given the specific environmental conditions. Ecdysone production by the PG has been shown to be circadian-gated and to respond to inputs from nutrition and tissue damage [3,5,40]. These inputs are relayed to the PG by neuropeptides produced by neurons that innervate the PG and by circulating peptide hormones that activate corresponding receptors on PG cells (figure 3). A study published by Siegmund & Korge [45] in 2001 described 11 sets of neurosecretory neurons that innervate the ring gland, and a recent study has provided additional information regarding the neuropeptides these express [46]. Of the 11 sets of neurons that target the broader ring gland, five were shown in the Siegmund & Korge study to directly innervate the PG [45]. The neurons that innervate the PG are presumed to secrete neuropeptides that activate corresponding receptors on PG cells, which could result in either activation or inhibition of ecdysone production and secretion. One set of neurons that is extremely well characterized is the prothoracicotropic hormone (PTTH) neurons, as will be discussed in the next section. Serotonergic neurons have also been found to directly innervate the PG to control ecdysone synthesis in response to nutrition, although these neurons do not produce neuropeptides, but rather the neurotransmitter serotonin [47]. However, the signals coming from some of the other neurons that innervate the PG remain uncharacterized.

In addition, peptide hormones secreted from elsewhere in the animal travel in the haemolymph and have the potential to bind to receptors on the PG to influence the production of ecdysone. Together, these circulating peptide hormones and the neuropeptides produced in neurons that innervate the PG suggest there is probably a rich and complex array of peptide regulators for ecdysone synthesis in *Drosophila*.

## 3. The first peptides shown to regulate ecdysone production were discovered in Lepidoptera

Early contributions to our knowledge of peptide control of ecdysone production came from studies in Lepidoptera. This is because these animals are typically larger than insects like *Drosophila*, which facilitates culturing of their prothoracic glands and also makes biochemical assays and purification of hormones more straightforward. Almost 100 years ago, Kopeć [48] described a brain-derived peptide responsible for regulating developmental transitions in the silkworm, *Bombyx mori*. After much work, this peptide, prothoracicotropic hormone (PTTH), was finally isolated from the silkworm in the 1970s (for review, see [49]). However, the molecular cloning and characterization of *Bombyx* PTTH did not occur until the early 1990s [50,51]. The release of PTTH from the brain in *Bombyx* was shown to correlate with an increase in ecdysone titres and developmental transitions [52–54], providing the first suggestion that PTTH stimulates the production of ecdysone.

The tobacco hornworm, *Manduca sexta,* has also been used as a model in which to study ecdysone production. An advantage of *Manduca* is that it has paired prothoracic glands which produce ecdysone at the same rate, which means that experimental manipulation can be conducted on one gland, with the other serving as a control [55]. Studies in *Manduca* found that PTTH was also synthesized in this species, but the peptide was produced in two different sizes; big PTTH and small PTTH [56,57]. Both of these peptides have been shown to stimulate *Manduca* prothoracic glands to produce ecdysone *in vitro* [58]. In *Manduca,* PTTH is produced in two lateral cells in the larval brain [59] and then moves to the CA where it stored and later released [60].

During efforts to biochemically purify PTTH from *Bombyx* brains [61] another peptide was identified [62,63]. While researchers were not able to demonstrate that it stimulated ecdysone production in *Bombyx*, this experiment was successful in another moth species, the saturniid moth, *Samia cynthia ricini* [64]. This peptide was named bombyxin and was later shown to share homology with vertebrate insulin [62,63,65]. This was the first demonstration of the presence of insulin-like peptides in invertebrates. Together, these studies on lepidopteran PTTH and bombyxin formed the basis of early investigations into the regulation of ecdysone synthesis in insects. However, the advanced molecular genetic approaches possible in *Drosophila* then provided the opportunity for detailed characterization of their roles, and the signalling pathways they activate.

### 3.1. Prothoracicotropic hormone is an important neuropeptide regulator of the *Drosophila* prothoracic glands

In a pivotal study, McBrayer *et al.* [3] showed that genetically ablating the PTTH-producing neurons that directly innervate the PG in *Drosophila* resulted in animals with a significant developmental delay and increased final body size. These phenotypes were found to be due to reduced expression levels of ecdysone biosynthesis genes and subsequent low ecdysone titres [3]. It was later shown that *PTTH*-null mutants also have a significant developmental delay, increased adult size and low ecdysone titres [66], further suggesting that PTTH does indeed regulate developmental timing by controlling ecdysone production. However, the developmental timing and body size defects seen in the PTTH null mutants were less severe than in animals where the PTTH-producing neurons had been ablated.

**Figure 4.**
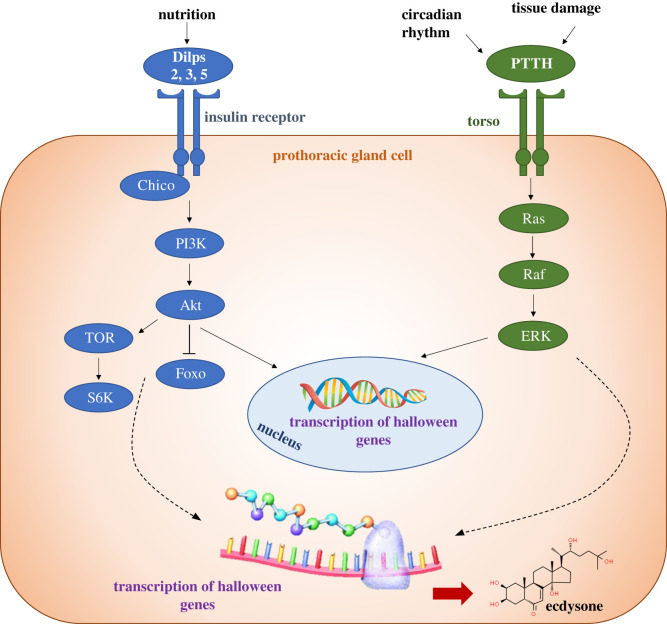
The PTTH/Tor and Dilp/InR pathways regulate ecdysone production in the *Drosophila* PG. Dilps 2, 3, and 5 are secreted in response to nutrition. They then bind to the Insulin Receptor (InR) which auto-phosphorylates. The insulin receptor substrate, Chico, then recruits Phosphoinositide 3-kinase (PI3 K) as well as other proteins and downstream effectors. PI3 K activates Protein Kinase B (Akt), which stimulates protein synthesis by activating downstream kinases Target of Rapamycin (TOR) and S6 Kinase (S6 K), and by inhibiting the transcription factor Forkhead Box O (FoxO) activity. This signalling cascade leads to the transcription and translation of ecdysone biosynthesis genes. Prothoracicotropic Hormone (PTTH) binds to Torso to trigger Ras/Raf/ERK signalling to drive the production of ecdysone, via either the transcription or translation of ecdysone biosynthesis genes (for review, see [68]).

In *Drosophila*, the receptor for PTTH is a receptor tyrosine kinase called Torso (Tor) [67]. Knockdown of *tor* specifically in the PG results in a significant developmental delay and an increase in final body size due to a prolonged third larval stage [67], phenocopying the ablation of PTTH neurons [3]. Upon binding PTTH, Tor triggers Ras/Raf/ERK signalling and this appears to regulate ecdysone biosynthesis at both the transcriptional and translational level [15,67] (figure 4). When PTTH-producing neurons are ablated in *Drosophila* several ecdysone biosynthesis genes show reduced expression [3]. These experiments thus suggest that the PTTH/Tor pathway regulates transcription of ecdysone biosynthetic genes. In addition, the PTTH/Tor pathway also appears to regulate the translation of these enzymes [15,67].

While it is known that the PTTH/Tor pathway triggers Ras/Raf/ERK signalling, it is unclear how exactly this pathway regulates the translation/transcription of the Halloween genes. The Ras/Raf/ERK cascade is known to activate a number of transcription factors via phosphorylation (for review see [69]). As several transcription factors have been identified as regulators of the Halloween genes [70–75], it is possible that components of the Ras/Raf/ERK cascade phosphorylate these transcription factors to activate them. The Ras/Raf/ERK cascade has been shown to also regulate protein translation via activating the ribosomal protein, S6 kinase [76]. However, it is unknown how PTTH/Tor signalling might specifically regulate translation of the Halloween genes.

Although it is widely accepted that PTTH is the key neuropeptide that triggers developmental maturation, evidence suggests additional timing signals are produced from the PTTH neurons. When the PTTH neurons are ablated a substantial proportion of animals are still able to undergo metamorphosis, albeit after a substantial delay [3]. In addition, flies that bear a null mutation for PTTH show delayed development, but are still viable [66]. In a recent study two additional peptides, *jelly belly* (*jeb*) and *PDGF- and VEGF-related factor 3* (*pvf3*), were shown to be expressed in PTTH-producing neurons [77]. When the individual receptors for *jeb* and *pvf3* are knocked down in the PG, animals exhibit a mild developmental delay and increased body size [77]. Interestingly, when the receptors for both *jeb* and *pvf3* are knocked down in the PG in a *ptth* null mutant background, the developmental delay is more prolonged, and there is a larger increase in body size than in animals where only the individual receptors for *jeb* and *pvf3* have been knocked down in the PG [77]. This suggests that Jeb and Pvf3 signalling interact with PTTH signalling in the PG to positively regulate ecdysone production. However, given that animals are still able to survive in the absence of PTTH, Jeb and Pvf3, these cannot be the only factors controlling ecdysone production.

PTTH production is known to be regulated in response to neuropeptides that are produced in other neurons which communicate with PTTH neurons. For example, the neuropeptide Corazonin (Crz) has been shown to regulate PTTH production in the PTTH neurons to control basal levels of ecdysone [78]. Crz neurons were found to make direct contact with and activate the PTTH neurons [78]. When the Corazonin receptor (*CrzR*) is knocked down in the PTTH neurons, or when *crz* is knocked down in Crz neurons, animals grow faster than controls during the L3 stage. This suggests that Crz negatively controls growth via inhibition of PTTH production [78]. Furthermore, the authors suggest that regulation of PTTH by Crz is nutrition dependent, as Crz-producing neurons receive information from other nutrient-sensing neurons.

It was also recently shown that the neuropeptide Allatostatin A (AstA) acts on the PTTH neurons to regulate PTTH secretion [79]. Knocking down either *AstA* in AstA-producing neurons, or its receptor, *AstA-R1,* in PTTH-producing neurons, results in a developmental delay [79]. Furthermore, knocking down *AstA-R1* in PTTH neurons results in the reduction of PTTH in the haemolymph, suggesting that AstA signalling induces this developmental delay by regulating PTTH secretion [79]. The AstA neurons and the PTTH neurons make contact with each other, suggesting that AstA is directly secreted onto PTTH neurons [79]. At present, it is unknown if AstA regulates PTTH secretion in response to a particular environmental cue.

Finally, in flies mutant for the neuropeptide *pigment dispersing factor* (*pdf*), the transcriptional profile of PTTH becomes altered, indicating that PDF influences *ptth* transcription [3]. PDF may achieve this effect by binding to the PDF receptor on PTTH neurons, as PDF-producing neurons have been shown to be in close proximity with PTTH-producing neurons [3]. However, a direct synaptic connection has not been demonstrated. It has also not yet been determined if the PDF receptor is expressed in the PTTH-producing neurons.

### 3.2. Insulin-like peptides regulate the function of the *Drosophila* prothoracic glands

After bombyxin was shown to regulate the production of ecdysone in moths [64], it was found that the *Drosophila* insulin-like peptides (Dilps) play this same role in *Drosophila* [5–7]. Eight Dilps, which are both functionally diverse and differentially expressed [80], have been described in *Drosophila*. Dilps 1–7 bind to the Insulin Receptor (InR), while Dilp8 binds to a different receptor and is more closely related to vertebrate relaxins [81–84]. Only *dilps 2, 3* and *5* are expressed in the insulin-producing cells (IPCs) in the CNS, and these peptides are secreted from these cells in response to nutrition [85]. Reducing Dilp secretion by the IPCs prolongs development time and results in small adults, indicating that one or more of Dilps 2, 3 and 5 play important roles in modulating ecdysone synthesis [86]. While the IPCs are in close proximity to the PG they do not innervate the PG and instead innvervate the CC [41]. Therefore, even if it is only a short distance, the Dilps must be secreted into the haemolymph by the IPCs and then travel to the PG to exert their function.

When the Dilps bind to InR it auto-phosphorylates and recruits Phosphoinositide 3-kinase (PI3 K) via the insulin receptor substrate, Chico (for review, see [87]). This promotes the activation of Protein Kinase B (Akt), which activates downstream kinases Target of Rapamycin (TOR) and S6 Kinase (S6 K) and ultimately inhibits the activity of the transcription factor Forkhead Box O (FoxO) (figure 4). At least some of the effects of insulin signalling on ecdysone synthesis are known to be mediated by the activity of FoxO [12]. FoxO exerts its effects, at least in part, by binding with a component of the ecdysone receptor, Usp to repress the expression of ecdysone synthesis genes [12].

During the third larval instar, the timing of the critical weight ecdysone pulse is known to be under the control of the insulin signalling pathway in the PG [5,12]. Upregulating the expression of FoxO significantly delays the timing of the critical weight ecdysone pulse [12], resulting in both an increase in body size and developmental delay [5–7]. The insulin signalling pathway has also been shown to affect endocycling in the PG cells at critical weight [88]. These endocycles are thought to play a critical role in the production of the pulse of ecdysone that induces the critical weight transition [88]. Whether endocycling is induced through the FoxO/Usp complex is unknown.

**Figure 5.**
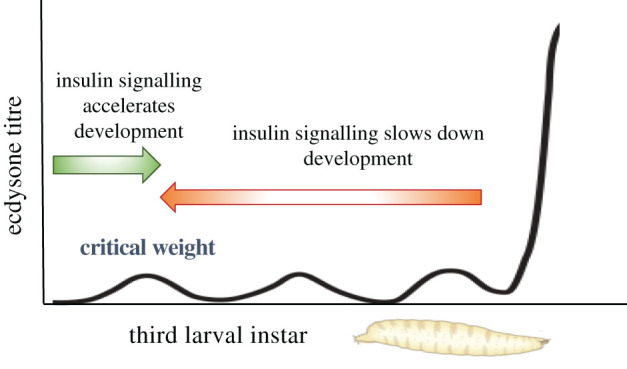
Insulin signalling plays different roles pre- and post-critical weight. Before critical weight is reached in the third larval instar, insulin signalling promotes developmental timing by positively influencing the production of ecdysone [5–7,13]. However, after critical weight has been reached, insulin signalling is thought to delay development by negatively influencing the production of ecdysone [13,89].

Interestingly, the insulin signalling pathway plays different roles in response to nutrition pre- and post-critical weight. In pre-critical weight larvae, once the appropriate nutrients have been acquired the insulin signalling pathway promotes the production of ecdysone [5–7,12,13]. By contrast, in post-critical weight animals, insulin signalling is thought to repress ecdysone production as starvation, which results in low insulin signalling, results in accelerated development [5,89]. This suggests that pre-critical weight, insulin signalling promotes ecdysone production, whereas post-critical weight, it has the opposite effect (figure 5). Correspondingly, differences have been observed in the ecdysone biosynthetic pathways that respond to InR activation. Early in the third instar, before critical weight, the insulin signalling pathway positively regulates mRNA levels of the Halloween genes, including *phm*, *dib, nvd, spok* and *sad* [6,7,12,15]. Conversely, later in the third larval instar insulin signalling in the PG appears to have no effect on mRNA levels of these same Halloween genes [15]. Instead of regulating the transcription of the Halloween genes, post-critical weight insulin signalling has been suggested to positively regulate translation of the Halloween genes Dib and Spok [15]. As post-critical weight insulin signalling is thought to negatively regulate ecdysone production, it is puzzling that it appears to positively regulate translation of Dib and Spok. It remains possible, however, that it negatively regulates translation of other Halloween genes post-critical weight, as they were not tested in this study.

It thus appears that insulin signalling may regulate the transcription of Halloween genes pre-critical weight, but also regulate the translation of some Halloween genes post-critical weight. However, more work is needed to fully describe the downstream signalling pathways activated in each case. Overall, these studies demonstrate the complex array of roles the insulin signalling pathway plays in regulating ecdysone synthesis in the PG, highlighting the importance of this pathway in controlling developmental timing.

An interesting question to ask is why might PG cells respond quite differently to InR activation at these different developmental stages? One possibility is that the different response to InR activation might arise from the length of ligand binding time. It has previously been shown that the length of time a ligand binds a receptor can affect its downstream responses [90,91]. For example, the mammalian Insulin Receptor activates different downstream pathways depending on whether it is bound to long-acting insulin, or short-acting insulin [92]. Therefore, it is possible that the length of time that the Dilps bind to InR determines whether the InR pathway positively or negatively regulates ecdysone production via modifying the activation of downstream signalling pathways. Similarly, it is possible that each of Dilps 2, 3 and 5 bind InR on the PG at different stages of development for different lengths of time, thereby triggering different downstream responses. In support of this, in cultured *Drosophila* cells it has been shown that Dilps 2 and 5 elicit different downstream effects upon binding to InR [93]. For example, Dilp 2 induces the phosphorylation of Akt only transiently while Dilp 5 induces sustained phosphorylation of Akt [93]. This would in turn effect the phosphorylation patterns, and therefore activation, of target genes further downstream.

## 4. Other peptide signalling pathways known to regulate ecdysone production in the *Drosophila* prothoracic glands

While the PTTH and Dilp signalling pathways are the best-characterized regulators of ecdysone production in the PG, evidence suggests that other pathways are also involved. In particular, when both the PTTH and insulin signalling pathways are downregulated in the PG (via the activin pathway), animals are able to survive up until the last larval stage [15]. This indicates that additional factors, other than PTTH and the Dilps, are required for at least the first two larval moults. Unsurprisingly then, a series of recent studies have uncovered several other peptide signalling pathways that directly regulate PG function. As well as some interesting examples of autocrine regulation (not covered here), several neuropeptides or peptide hormones secreted from other tissues have been implicated in regulating PG function via binding to their receptors on the PG. This is usually thought to occur in response to an environmental cue.

For example, Lgr3, a relaxin receptor which belongs to the larger family of leucine-rich repeat-containing G-protein coupled receptors (LGRs) [94], has been shown to regulate ecdysone production. The ligand for Lgr3, Dilp8, stimulates Lgr3 activity both *in vitro* [83] and *in vivo* [81,83]. Dilp8 is secreted by damaged imaginal discs during larval development to delay the development and to slow down the growth in undamaged discs [81,82,84]. This delay is important, as it gives damaged imaginal discs time to regenerate before metamorphosis. Dilp8 activates Lgr3 signalling in the PG to cause a reduction in basal, between-pulse, levels of ecdysone synthesis [40,95,96]. Because imaginal discs rely on ecdysone for their growth [97,98], the reduction of these basal ecdysone titres causes the inhibition of growth in undamaged imaginal discs [40,96]. This demonstrates how a peptide hormone secreted from elsewhere in the animal is able to directly elicit changes in PG function in response to an environmental cue, in this case, imaginal disc damage. Interestingly, Dilp8 plays a second role in regulating PG function as it also activates Lgr3 in a bilateral pair of neurons that make direct contact with the PTTH neurons. In so doing it inhibits PTTH secretion and results in delays in the timing of ecdysone pulses [81,83,84,99]. As well as regulating the PG directly, Dilp8 thus also represents an additional regulator of PTTH neuron function to those mentioned earlier.

Neuropeptide F signalling was also recently shown to regulate ecdysone production in the PG [100]. When the receptor for neuropeptide F, *NPFR*, is knocked down specifically in the PG animals have reduced ecdysone titres and reduced expression of the Halloween genes, *phm* and *dib*. NPFR signalling appears to exert its effects on ecdysone production by negatively regulating the insulin signalling pathway in the PG, as when *NPFR* is knocked down in the PG, insulin signalling activity is increased. In the larva, NPF is known to be expressed in the midgut [101]. NPF has not been shown to be expressed in neurons that innervate the PG nor in neurons that synapse with PTTH neurons, although this is difficult to rule out. As NPF has been shown to be able to act systemically in adult *Drosophila* [102], it is possible that it is secreted into the haemolymph in response to nutrition and then acts on the PG to influence ecdysone production.

Another peptide receptor shown to influence developmental timing is *Lgr1,* another member of the LGRs. Ubiquitous knockdown of *Lgr1* results in the suppression of puparium formation, reduced ecdysone levels and reduced expression levels of the ecdysone biosynthesis genes, *shadow* and *spookier* [103]. The suggested ligands for Lgr1 are Heterodimeric fly glycoprotein hormone-alpha2 (GPA2) and glycoprotein hormone-beta5 (GPB5) [104]. As it has not yet been elucidated, if Lgr1 is expressed in the PG it is unclear if it is involved in direct or indirect regulation of PG function.

### 4.1. Other peptide/receptor signalling pathways in the *Drosophila* prothoracic glands probably remain to be discovered

There are several indications that additional peptide hormone and neuropeptides that regulate the PG remain to be discovered. As well as the paired bilateral PTTH-producing neurons that innervate the PG, Siegmund & Korge [45] also identified ten other groups of neurons that innervated the ring gland. Five of these directly innervate the PG, suggesting that other neuropeptides produced in these neurons could act to regulate ecdysone synthesis. The neuropeptides produced by these neurons remain to be discovered, although could potentially include NPF, GPA2 and GPB5. It will be interesting to see if the rapid progress being made in single-cell approaches leads to the future characterization of the molecular profiles of these neurons, together with the standard expression studies of peptide hormones and their receptors.

In addition, while the signalling pathways triggered by photoperiod, tissue damage and nutrition have been at least partly uncovered [5,40,66,84,105], there are also other environmental factors known to regulate developmental timing. These include oxygen, temperature and larval density [8,9,106,107]. Presumably, these additional environmental conditions also elicit their effects on developmental timing by altering the production of ecdysone; however, the underlying molecular pathways which relay these types of environmental information to the PG are unknown.

Finally, studies in other insects also provide indications that additional peptide signalling pathways might function in the *Drosophila* PG. For example, in *Bombyx,* several other neuropeptides and receptors have been found to both positively and negatively influence ecdysone biosynthesis (table 1). Furthermore, in the kissing bug, *Rhodnius prolixus*, several other neuropeptide receptors have found to be expressed in the PG, all of which have *Drosophila* homologues (table 1). Given the significant commonalities observed so far across insects in many (but by no means all) aspects of the regulation of ecdysone, it is certainly possible that these peptides and receptors may play a role in the *Drosophila* PG. Given the progress recently made, in particular by genetic screening approaches, more pathways are likely to be described in the near future. The field will then need to turn its attention to the complex ways these pathways may interact in the PG to integrate responses to different regulatory cues, a challenging task indeed.

**Table 1.** Examples of other insect peptide/receptor systems known to function in or be expressed in the PG.

receptor	ligand	species	*Drosophila* receptor homologue	known function or expression pattern	reference
*Bombyx* neuropeptide GPCR-B2 (BNGR-R2)	pigment dispersing factor (PDF)	*Bombyx mori*	PDFR	stimulates ecdysone synthesis *in vitro*	[108]
unknown	orcokinins	*Bombyx mori*	unknown	stimulate ecdysone synthesis *in vitro*	[109]
diapause hormone receptor (DHR)	diapause hormone (DH)	*Bombyx mori*	none	expressed in PG and regulates ecdysone	[110]
neuropeptide receptor A34	unknown	*Bombyx mori*	CG30340	enriched in PG	[111]
corazonin receptor (CrzR)	corazonin (Crz)	*Rhodnius prolixus*	CrzR	expressed in PG	[112]
calcitonin-like diuretic hormone Receptor 1 (CT/DH-21)	unknown	*Rhodnius prolixus*	Hec	expressed in PG	[113]
calcitonin-like diuretic hormone receptor 1 (CT/DH-R1)	diuretic hormone 31 (Dh31)	*Rhodnius prolixus*	Dh31R	expressed in PG	[113]
bommo-myosuppressin receptor (BMSR)	bommo-myosuppressin (BMS) and bommo-FMRFamides (BRFas)	*Bombyx mori*	MsR1 and MsR2	suppress ecdysone synthesis	[114,115]
sex peptide receptor (SPR)	prothoracico-static peptide (PTSP)	*Bombyx mori*	SPR	suppress ecdysone synthesis	[116]
